# To the Editors of the Medical and Physical Journal

**Published:** 1804-05-01

**Authors:** G. N. Hill

**Affiliations:** Chester


### *To the Editors of the Medical and Physical Journal.*

Gentlemen,

I Am sorry indisposition prevented me from replying to Mr. Ward's statement in your January Number, in the succeeding month. Intentional misrepresentation nothing can justify; being unconscious of the slightest design of this kind throughout all I have written on the subject of opium, I certainly deem no further apology necessary. I have, from Mr. Ward's first promulgating his thoughts on the matter (viz. in 1799) thought, and still continue to think, that there was a time when he deemed opium a tonic stimulant. It appeared evident to me from his early reasoning, and from his mode of practice. And is there any thing blameworthy that he, or any practitioner, should, from good premises, change their opinion? Do we any of us hold exactly the same opinion of every medicine in 1804 as we did in 1784? The full passage on which Mr. W. lays so much stress, as misquoted and injuriously misrepresented, runs thus: Vol. 6, p. 480—"Various cases have been recorded where it was thus introduced (opium by friction), and was found to exert its salutary virtues, so as to produce the most beneficial consequences (and, without occasioning those inconveniences which often arise from its internal use, especially in large doses), after the same medicine both alone and joined with OTHER antispasmodics, tonics, &c." Now I confess my stupidity in still being unable to view this passage in any other light than as a classification of opium with antispasmodics and tonics. Your Readers will judge for themselves; but to put the matter out of doubt, in my humble apprehension, nothing more is requisite than to give a short quotation from the P. S. to your 50th No. where Mr. W. says—"and that my choice was not at all directed by any opinion I had formed of the modus operandi of opium (for I confess I had not then paid sufficient attention to the subject to enable me to make up my mind upon it)." Thus Mr. W. acknowledges all for which I contend, after having held the subject before the public near five years. I can truly say, "Odi omnes in scribendo acerbitates." I never was ambitious of the particular notice of Mr. W.; I wrote what your indulgence has already inserted in my desultory manner for the public, well knowing its candour, and that medical novices particularly have never failed to meet that candour when their their designs have clearly appeared to be the public good. Mr. W. long since claimed a right to be heard out, and then to be answered. In compliance with this claim, the Editors of the Journal suppressed the paper I sent them in April, 1803, till December. In November the public was favoured with Mr. W.'s case of cancer (still unconcluded) preliminary to the recital of which he expresses disappointment at his proposed definitions not being noticed, amended, or his opponent's sentiments given on so interesting a topic; and all this after requesting their silence until he could say, "and this is the conclusion of the whole matter." On perusing the paper upon cancer, I wrote a short one, containing some thoughts thereon, so far as opium was concerned, and on what I had before advanced respecting Mr. W's unsettled opinion, which, if you will indulge me by inserting now, will convince Mr. W. I had not been inattentive to his request in No. 50. I should instantly have cleared up this matter in April, had I not concluded my paper of that date would have been sufficient. I have already said I hate controversy; I have no ambition of this sort, but I will yield to no man, not even Mr. W. in my ambition to do good, in my endeavours to produce something practically useful to mankind. Should I ever gain this proud laurel of the medical man, my desultoriness will be pardoned, though I must add, it ill becomes an author to comment on such a fault, who has already taken nearly five years to explain his theory, and written near an hundred pages on the subject; and after all this, has not been willing, with all his time and talent, to attempt a refutation of one practical fact out of numbers adduced to disprove the hypothesis he has given to the public.

*Chester, March* 8, 1804. I am, &c. G. N. HILL.

